# Supplementary material for: Bioaffinity-based surface immobilization of antibodies to capture endothelial colony-forming cells
Source: PLoS One. 2022 Aug 30;17(8):e0269316. doi: 10.1371/journal.pone.0269316 (PMC9426933; doi:10.1371/journal.pone.0269316)
Supplement: S1 Appendix — (PDF) [file pone.0269316.s001.pdf]

APPENDIX S1

Bioaffinity-based surface immobilization of antibodies to capture endothelial colony-forming cells

Mariève D. Boulanger[1*], Hugo A. Level[1*], Mohamed A. Elkhodiry[1*], Omar S. Bashth[1], Pascale Chevallier[2,3], Gaétan Laroche[2,3], Corinne A. Hoesli[1Ψ]

[1] Department of Chemical Engineering, McGill University, Canada.

[2] Laboratoire d'Ingénierie de Surface, Centre de Recherche sur les Matériaux Avancés, Département de Génie des Mines, de la Métallurgie et des Matériaux, Université Laval, Québec, Canada.

[3]Centre de Recherche du Centre Hospitalier Universitaire de Québec, Hôpital St-François d'Assise, Québec, Canada.

*Equal contribution

[Ψ]Corresponding author

Supplemental Methods

1. **Detection of Amines via the Orange II Assay**

The surface concentration of primary amines was quantified using the Orange II assay (1, 2). The Orange II dye has a negatively charged sulfonated group that can preferentially bind to positively charged protonated primary amines in an acidic solution. The dye can then be released by adjusting the pH and then colorimetrically quantified. Surfaces cut into 1 cm^2 surface-modified polystyrene pieces were transferred into 10 mL polystyrene tubes (#T406-2, Simport scientific, Beloeil, Ca). The samples were then submerged in Orange II sodium salt solution (14 mg/mL Orange II, 75370, Sigma Aldrich in RO water adjusted to pH 3 with 37% HCl; 1.5 mL added per tube). After incubating for 30 min at 40°C, samples were rinsed with the acidic solution to remove all unbound dye and air-dried before being immersed in 1 mL of alkaline solution (RO water adjusted pH to 12 with 5N NaOH solution) to desorb the dye. The pH of the desorbed dye solution was then readjusted to a pH of 3 by adding 1% v/v of 37% HCl to each tube. All solutions were transferred to a cuvette and absorbance measurements were taken at 484 nm on a Genova spectrophotometer (Jenway, Staffordshire, UK). Primary amines were quantified by comparing the absorbance obtained to a standard curve generated by adding Orange II dye in acidic solution at known concentrations ranging between 0.3 µg/mL and 140 µg/mL.

2. **Contact Angle Measurements**

The contact angles between deionized RO water and functionalized surfaces were measured by the sessile drop method using an OCA 150 system (DataPhysics Instruments GmbH, Filderstadt). Water drops of 5 µL were deposited at a rate of 0.5 µL/s onto Purecoat ™ substrates, with or without S-SMPB treatment. Images of the drops in contact with surfaces captured at the end of drop spreading were recorded. The average between the left and the right static contact angle values were determined for each image using the SCA-20 software (DataPhysics Instruments).

3. **Atomic Force Microscopy (AFM) imaging of modified polystyrene**

A commercial aminated petri dish was cut into 1 cm*1 cm coupons and then functionalized according to the protocol previously described. Briefly, the coupons were covered with 3mg/mL Sulfo-SMPB for

2 hours, rinsed with PBS and then reacted with 5.5 uM Cys-Protein G. Primary antibodies (anti-CD309 mouse IgG1) were then immobilized on the surface at 5 µg/mL for 1 hour. The surfaces were finally rinsed twice with PBS and RO water before air drying and AFM imaging. An unmodified aminated polystyrene coupon was analyzed as a control. The images were acquired both in ambient conditions using a NanoscopeV-Dimension ICON atomic force microscope (Bruker, Santa Barbara, CA, USA). All Imaging was done in the PeakForce Tapping mode (PeakForce QNM®) using *SCANASYST-Fluid* aluminum-coated Silicon Nitride probes with tip radius ranging between 2-10 nm and a nominal spring constant of 0.4 N/m. The scanning rate was 1 Hz and for each condition, two images were taken with two different resolutions, 5 µm x 5 µm and 1 µm x 1 µm.

Image treatment and analysis was performed with the NanoScope software (Bruker). The arithmetic roughness average was computed for the 1 um2 images to avoid the surface defects observed at larger scales. The depth distribution of a 500 nm x 500 nm area was also obtained for each coupon.

Supplementary Figures

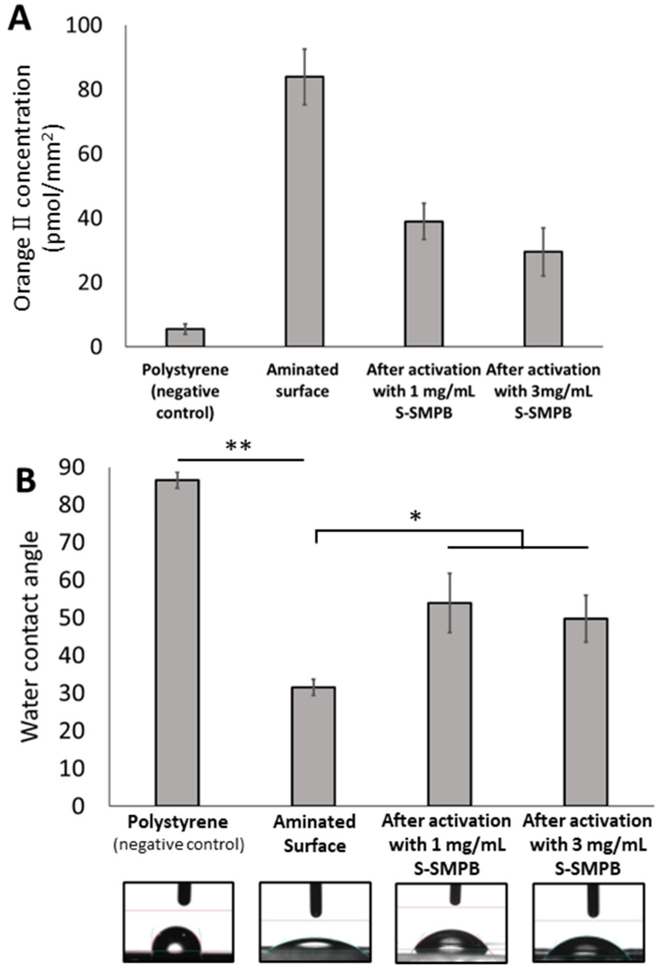

Figure A1. Surface characterization before and after S-SMPB treatment. (A) Amino quantification of substrates using Orange II dye before and after activation with S-SMPB. Untreated polystyrene was used as a negative control. **(B)** Water contact angle on aminated polystyrene substrates before and after activation with S-SMPB. S-SMPB was applied at 1 mg/mL and 3 mg/mL prior to rinsing, drying and goniometry. *P < 0.05; **P < 0.01 with N=3 experimental replicates on independent surfaces. For water contact angle measurements, the reported values represent the average ± the standard error of the mean of 10 images per surface from 3 independent replicas.

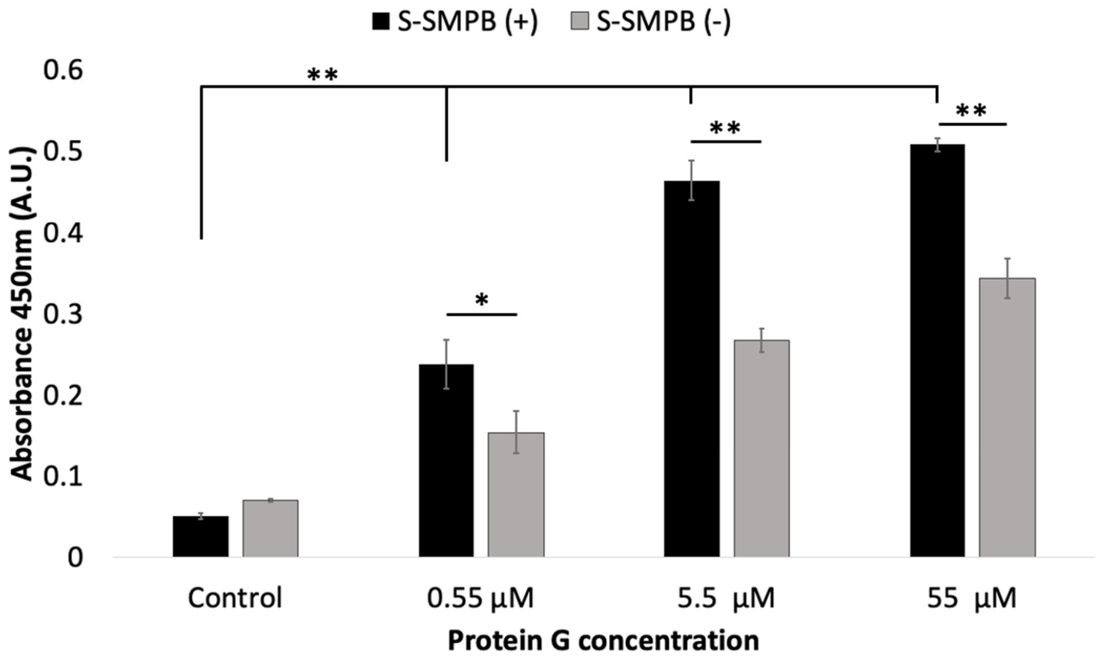

Figure A2. Direct ELISA detection of surface-immobilized protein G after covalent conjugation or adsorption, performed on a different aminated substrate (amine treated 96-well plate, #MCB02F-AM1, Biomat, Santa Margherita, Italy). Control: no protein G added. **P < 0.01, *P < 0.05 with N = 3.

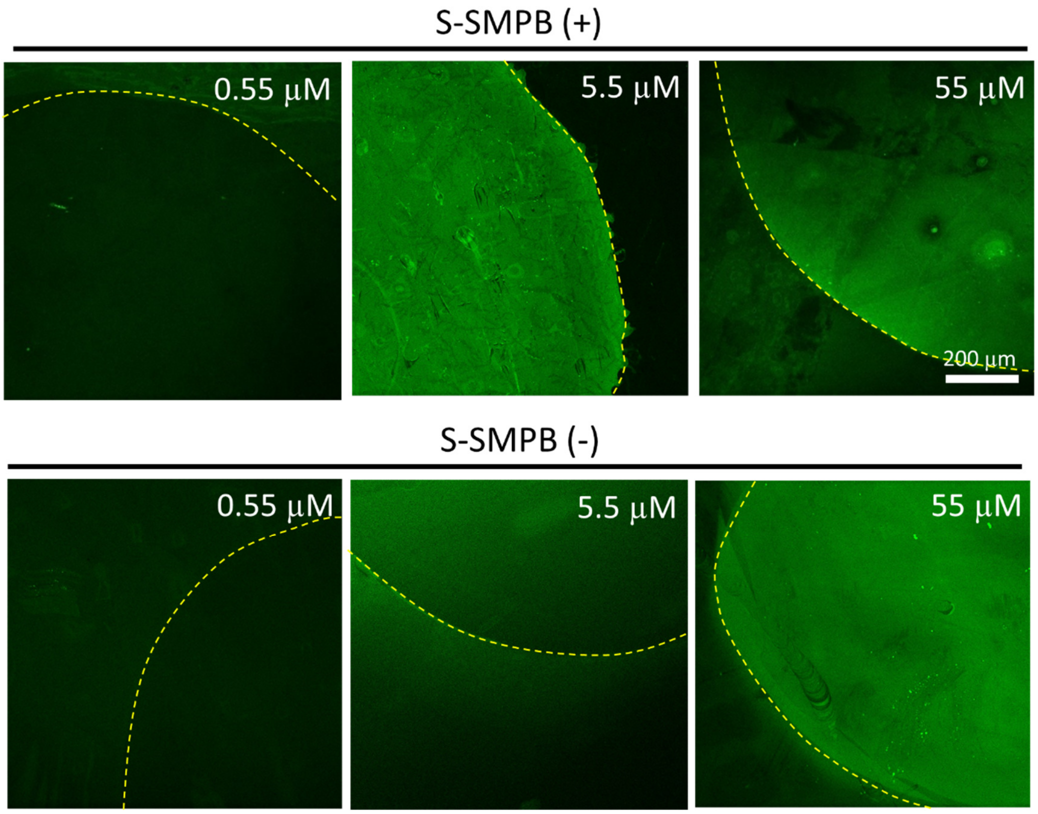

Figure A3. Effect of protein G polypeptide concentration in solution on anti-CD31 immobilization via conjugated vs adsorbed protein G. PureCoat^{TM} aminated surfaces were functionalized with protein G polypeptides either via covalent conjugation (S-SMPB(+)) or adsorption (S-SMPB(-)). The protein G polypeptides were applied at different concentrations in solution as indicated in white text on each panel. Mouse IgG1 anti-CD31 antibodies were then added and detected through fluorophore-labelled anti-mouse secondary antibodies. Each condition was applied at least 3 times. Representative spots are shown. Dotted yellow lines represent spot contours.

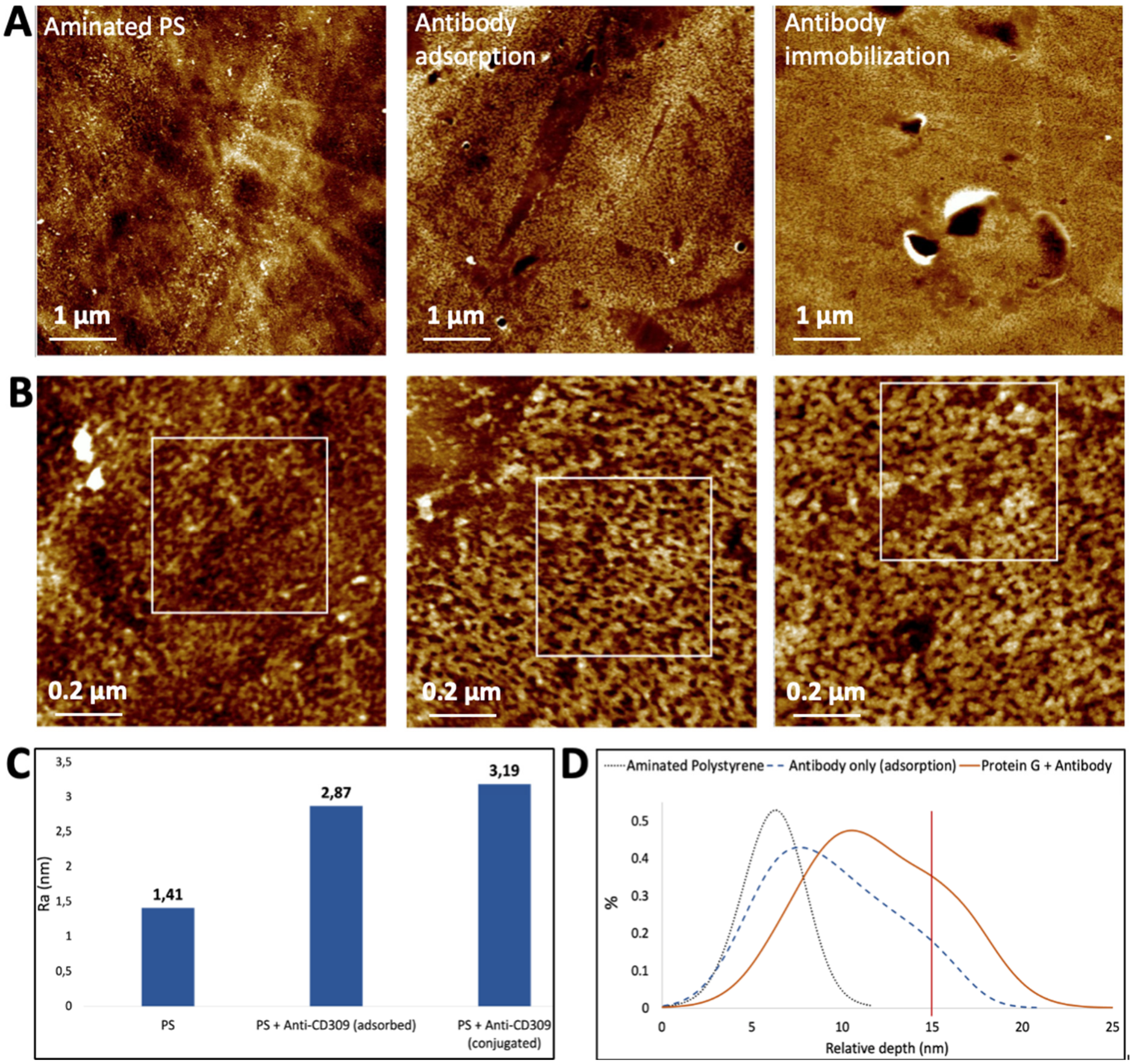

Figure A4. AFM imaging of modified polystyrene surfaces. **(A)** 5 um^2 images of an unmodified polystyrene coupon (aminated PureCoat^{TM} amine), antibody adsorption (surface functionalization omitting S-SMPB) and anti-CD309 antibody immobilization on protein G (complete functionalization scheme). **(B)** 1 um^2 images of the same conditions. The white squares represent the 500 nm^2 areas that were used to obtain the surface depth histograms. **(C)** Surface roughness (arithmetic average, Ra) obtained from the 1 um2 images to avoid surface defects visible at higher scales. **(D)** Depth distribution profiles obtained from the raw height data of 500 nm^2 areas on each image. The vertical line (% Hist) represents a fitted histogram of the distribution frequency. The vertical red line at 15 nm marks the typical length of an antibody.

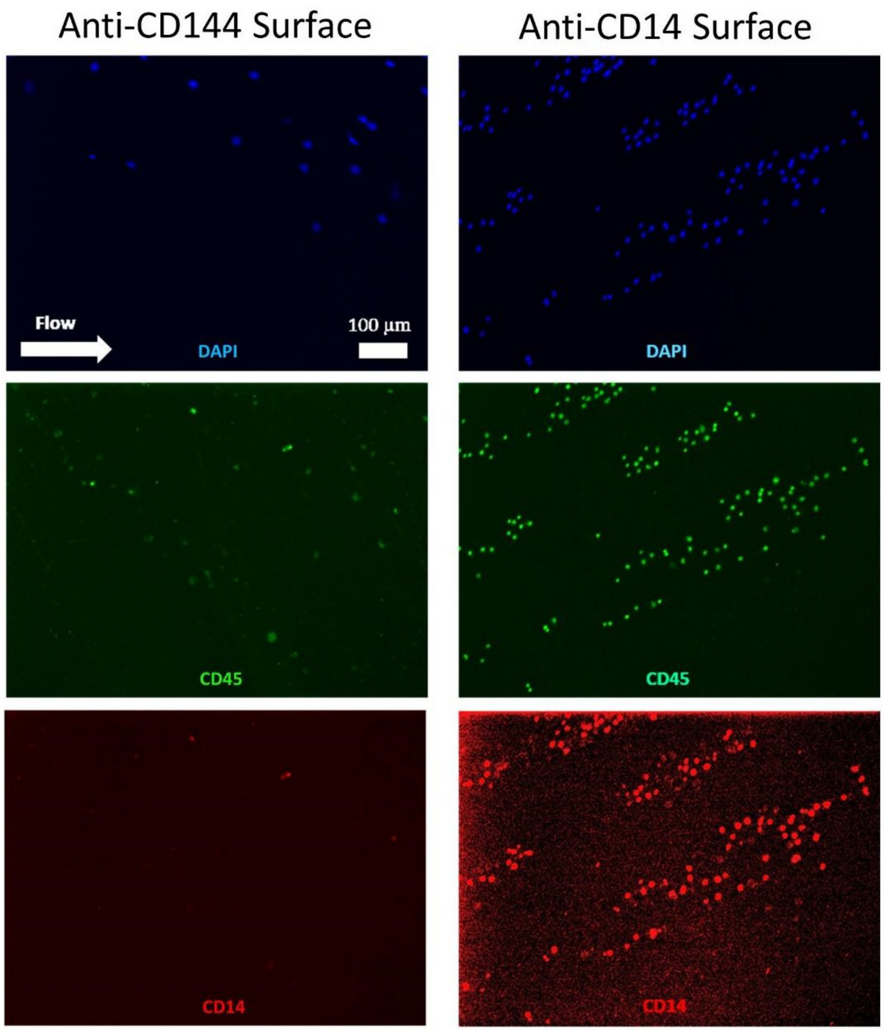

Figure A5. Representative images of peripheral blood mononuclear cells captured from flow on surfaces with anti-CD144 or anti-CD14 antibodies immobilized via conjugated protein G polypeptide. Nuclei (blue) were stained with DAPI, CD45 hematopoietic marker was detected with a rabbit anti-CD45 primary antibody and an AF488 goat anti-rabbit secondary antibody, and CD14 monocyte marker was detected with a mouse anti-CD14 primary antibody and an AF555 goat anti-mouse secondary antibody.

Supplementary References

1. Noel S, Liberelle B, Robitaille L, De Crescenzo G. Quantification of primary amine groups available for subsequent biofunctionalization of polymer surfaces. Bioconjugate chemistry. 2011;22(8):1690-9.

2. Uchida E, Uyama Y, Ikada Y. Sorption of low-molecular-weight anions into thin polycation layers grafted onto a film. Langmuir. 1993;9(4):1121-4.
