# Supplementary material for: Bioaffinity-based surface immobilization of antibodies to capture endothelial colony-forming cells
Source: PLoS One. 2022 Aug 30;17(8):e0269316. doi: 10.1371/journal.pone.0269316 (PMC9426933; doi:10.1371/journal.pone.0269316)
Supplement: Attachment — Submitted filename: Paper Review_ECFC Capture with antibody coated surfaces.docx [file pone.0269316.s004.docx]

Review

Overall Opinion

- The paper is written to a high grade and is an eloquently designed study with a detailed methodology and results section.

Overall Comments

- Could you use this technology be adapted to isolate ECFCs from peripheral blood for in vitro expansion as this is often a problem due to the low frequency of ECFCs in PBMCs?
- Is there any evidence to support the idea that ECFCs can proliferate successfully on this novel surface?
- There is a lot of review articles references, the inclusion of a higher number of primary articles is needed.
- It may be worth having a chemical diagram to demonstrate the process + binding (even if it is part of the supplemental material) such as that featured in the following paper, figure 1, which was previously published by your lab. It would diagrammatically demonstrate the binding to the aminated surfaces. <https://pubs.rsc.org/en/content/articlepdf/2020/bm/d0bm00650e?page=search> Bashth, O.S., Elkhodiry, M.A., Laroche, G. and Hoesli, C.A., 2020. Surface grafting of Fc-binding peptides as a simple platform to immobilize and identify antibodies that selectively capture circulating endothelial progenitor cells. Biomaterials Science, 8(19), pp.5465-5475.

Abstract:

- Authors should mention that their strategy facilitates improved ECFC binding due to improved orientation of capture antibodies.

Introduction:

- Background well described. Each point is explained and referenced before making the next point. However many of the references used are review articles and not primary articles, it would be beneficial to include more primary references for points made if possible
- Line 41: ‘Capturing EPCs, … ‘ should include primary references such as: <https://ashpublications.org/blood/article/104/9/2752/19414/Identification-of-a-novel-hierarchy-of-endothelial> Ingram, D.A., Mead, L.E., Tanaka, H., Meade, V., Fenoglio, A., Mortell, K., Pollok, K., Ferkowicz, M.J., Gilley, D. and Yoder, M.C., 2004. Identification of a novel hierarchy of endothelial progenitor cells using human peripheral and umbilical cord blood. Blood, 104(9), pp.2752-2760.
- Line 64: What do the authors mean by ‘partial denaturation’ and is this the appropriate term in this case?
- Line 60: ‘Using passive adsorption, …’ may benefit from referring to a primary article along with the already referenced to better support the point being made
- Line 80, 82: Due to the confusion associated with EPCs vs ECFCs that exists in the literature I think it important to keep it consistent after introducing ECFCs, therefore I suggest using ECFCs throughout the manuscript.

Methods:

- Methods section is very well described with detailed description of step by step procedures
- Line 104: minor typo of ‘2N’ where I assume it should be ‘2M’
- Line 106: The authors mention that the antibody coated plates were left to dry for up to one week, is there any idea as to whether there is any degree of antibody denaturation or reduced cell capture with time?
- Line 159: How did the authors confirm that only the FAB part of the chick antibody binds to the protein G?
- Section 2.6: Why was the surface not rinsed with BSA to prevent antibody non-specific binding? There seems to be quite a bit of background fluorescence in the images outside the drop area where there should be no protein G to the point where there is more fluorescence in the background than the lowest dose of protein G.
- There is no evidence that the data was tested for normality.
- For improved reproducibility/comparibility it would be helpful if the authors provided the ECFC donors mean age, N number and genders

Results

- Figure 2)
  - Why were only the 1mg/ml and the 3mg/ml included in part B (Orange II concentration) and not 0.5 and 1.5mg/ml treated surfaces?
  - There is no explanation as to why 1mg/ml and 3mg/ml were used going forward. Or which one was used for future studies
- Paragraph starting on line 251: Is it possible that S-SMPB is binding more than 1 protein G which is facilitating the higher absorbance?
- Figure 4)
  - What S-SMPB concentration was used for this experiment?
- Figure 5)
  - Is there more protein G binding outside the drop in the 0.55uM concentration? (Figure Bi) Can the authors suggest a reason for why it would be higher?
  - The inclusion of a S-SMPB(-) fluorescence image here as a negative control would also benefit to highlight the improvement in fluorescent signal
  - Why continue with CD144 and not CD31? Since the authors decided to use CD144 for the remainder of the study then the images in figure 5B should be of CD144 and not CD31.
    - Additionally, the authors should include fluorescent images of the other antibodies mentioned in section 3.3 in the supplementary figures section (i.e. anti-CD105, anti-CD144, CD14).
  - There are some ‘defects’ in binding in some areas of the droplet resulting in non-homogenous staining, especially in the higher concentrations, and therefore would indicate inhomogeneity in ECFC binding later. Would this affect endothelialisation of a graft with ECFCs if there are regions of non-binding?
  - What concentration of protein G was used to generate the graph in part C? and why was this concentration chosen over the others?
- Section 3.4:
  - The authors have not explained their reasoning for selecting CD144 over CD31 and CD105 as a capture antibody?
  - While I see that using ECFCs at a low shear stress demonstrates an important initial proof of concept of cell capture, I believe an important experiment that would need consideration is passing PBMCs over the treated surface to determine ECFC capture from a mixed cell population and at rate at a higher and more physiological shear stress for longer periods of time. i.e. to better mimic in vivo conditions and to demonstrate proof of concept in a more relevant in vitro model. This would also better facilitate moving into an animal model. Additionally, due to the potential inflammatory effect of the protein G this experiment could also double up to demonstrate immune cell activation.
- Figure 6)
  - Interesting that the ECFCs indicated by the green triangle seem to have lower binding than the others in all conditions. It might be worthwhile measuring CD144 expression on the ECFCs of that donor relative to the others.
- Figure S1
  - Was there any endothelial specific marker (not CD144) used to identify any ECFC binding to the surface? It will be problematic if only immune cells are binding to the surface, this may prevent any ECFCs binding to the surface.

Discussion

- While the discussion discusses the feasibility of the strategy used in this paper and compares it to a past paper providing a balanced discussion, it does not compare it to other modification strategies used by other papers/groups. An improved literature comparison would benefit this section.
- Line 324: Due to the confusion that remains in the field it may be best to consistently use the term ‘ECFC’ instead of ‘EPC’ as EPC can be thought of as a cell with immune cell properties.
- Line 355: Change EPC to ECFC

Conclusion

- Line 360: Change ‘endothelial progenitor cell’ to ‘ECFC’
- Line 369: Change ‘EPC’ to ‘ECFC’
